# Saved His Friends and Died

**Published:** 1893-03

**Authors:** 


### SAVED HIS FRIENDS AND DIED.

The story of Sogoro, a peasant of Japan, is one of the most pathetic in the annals of heroism. In 1644, the country folk of Sakura were so oppressed by land agents that their condition appeared to them simply unbearable. They had no newspaper to set forth their wrongs, and remonstrance of any sort was dangerous. Driven to desperation some of them met together and prepared a petition to the Daimio, who was spending in dissipation at Yedo the money wrung from them by taxation.

They wrote and sent the petition, but no notice whatever was taken of it. Possibly no one had ever taken the trouble to read it, and their wrongs seemed to be without remedy.

Moved by the general suffering, Sogoro, a man of middle age, determined, as a last and desperate resort, to present the petition in person to his August Greatness, the Tycoon. Taking leave of his friends, he went to Yedo, secreted himself under a bridge which the great man was to pass, and at the right moment pushed the petition at the end of a long bamboo directly into the royal hands.

The act was without parallel in all the history of Japan. A mere peasant had disturbed the royal seclusion, and at the same moment broken the etiquette of the realm into a thousand pieces. The enormity of the act led to immediate inquiries into the circumstances of the case, and the justice of the complaint was fully proven.

The peasants' wrongs were at once redressed, but since decorum must be preserved in Japan, at any cost, the one man who had thus served his people was delivered over for punishment to the very Daimio of whom he had complained.

By his order Sogoro, his wife and their three children were put to death. To-day a monument marks the spot where they died, and their names are held in grateful remembrance.

